# Socioeconomic inequalities in infant mortality in Colombia: a nationwide cohort study during 10 years

**DOI:** 10.1136/bmjgh-2024-018526

**Published:** 2025-08-21

**Authors:** Giancarlo Buitrago, Carol Guarnizo-Herreño, Javier Eslava-Schmalbach

**Affiliations:** 1 Universidad Nacional de Colombia, Bogota, Colombia; 2 Fundación Cardioinfantil Instituto de Cardiología, Bogota, Colombia; 3 Hospital Universitario Nacional de Colombia, Bogota, Colombia

**Keywords:** Global Health, Health policy, Health economics, Cohort study, Paediatrics

## Abstract

**Background:**

Although socioeconomic inequalities in infant mortality are well-documented globally, there is limited evidence from longitudinal studies, particularly in low-income and middle-income countries. This study investigates the association between maternal socioeconomic conditions, health system affiliation and infant mortality, and it measures the related socioeconomic inequalities in Colombia over a decade.

**Methods:**

A retrospective cohort study was conducted using data from the Unified Affiliation Registry on all singleton live births in Colombia from 1 January 2011 to 31 December 2020. Birth and death records were linked using anonymised maternal IDs. The primary outcome was 1-year survival. Key exposures included maternal educational level, health system enrolment scheme and area of residence. Cox regression models were used to estimate HRs, adjusting for relevant covariates. The Relative Index of Inequality (RII) and the Slope Index of Inequality (SII) were also calculated.

**Findings:**

Among the 5 951 953 live births recorded, 5 605 111 were analysed. Significant inequalities were noted based on maternal education, health system affiliation and area of residence. Children of mothers with primary or lower education had a 50% higher risk of dying in the first year (adjusted HR (aHR) 1.50; 95% CI 1.44 to 1.56). Those with uninsured mothers had a 61% higher risk compared with those with mothers in the contributory health scheme (aHR 1.61; 95% CI 1.54 to 1.68). Additionally, children of mothers in dispersed rural areas had an 8% higher risk of first-year mortality compared with those in urban municipal centres (aHR 1.08; 95% CI 1.05 to 1.11). There was a clear pattern of social gradients in infant mortality (educational level: RII 1.55, 95% CI 1.49 to 1.62, and SII 4.12, 95% CI 3.76 to 4.48; health insurance scheme: RII 1.67, 95% CI 1.60 to 1.73 and SII 4.78, 95% CI 4.42 to 5.14; residence’s zone: RII 1.12, 95% CI 1.07 to 1.17 and 1.04, 95% CI 0.63 to 1.44) and a notable increasing trend in educational inequalities (The RII and SII revealed increasing from 2011 to 2020 (RII from 1.18 to 1.92; SII from 1.59 to 5.74)).

**Interpretation:**

Despite Colombia’s economic growth, the persistence and increase of socioeconomic inequalities in infant mortality found in this analysis highlight the need for comprehensive policy reforms targeting healthcare access and socioeconomic conditions.

**WHAT IS ALREADY KNOWN ON THIS TOPIC**

- Socioeconomic inequalities are well-documented determinants of infant mortality, particularly in low-income and middle-income countries (LMICs). Previous studies have primarily relied on survey data or aggregate statistics, which limit the ability to evaluate longitudinal survival outcomes and their association with maternal sociodemographic characteristics.

**WHAT THIS STUDY ADDS**

- This is the first nationwide study from an LMIC to link individual-level birth and death certificates over a 10-year period, enabling precise estimation of 1-year survival for all live births in Colombia. The findings reveal significant inequalities in infant mortality by maternal education, health insurance scheme and area of residence. Moreover, they show that while overall infant mortality has declined, this progress has been largely concentrated among socioeconomically advantaged groups—especially mothers with higher education and those insured with the health system—resulting in a widening equity gap.

**HOW THIS STUDY MIGHT AFFECT RESEARCH, PRACTICE OR POLICY**

- This study sets a precedent for similar research in other LMICs by demonstrating the feasibility and value of using administrative data to analyse health inequalities. Its findings underscore the urgent need for targeted public policies that address the structural determinants of child health, emphasising improvements in maternal education and equitable access to healthcare to reduce infant mortality.

## Introduction

 Socioeconomic inequalities significantly impact child health outcomes worldwide, contributing to differences in infant mortality, morbidity and overall health status.^1^ Children born into lower socioeconomic conditions are at higher risk of experiencing adverse health outcomes, which can affect their development and long-term health.^2 3^ Such disparities often manifest in higher rates of preterm births, low birth weights and infant mortality among disadvantaged populations, emphasising the need for public policies targeting the known structural determinants of child health together with targeted public health interventions.

Socioeconomic inequalities have increased and have been studied deeply over the past 40 years.^4 5^ These disparities have profound implications for health outcomes, as evidenced by numerous studies since the 1980s that highlight the role of occupation and the hierarchical effect of socioeconomic status on health outcomes. One of the most significant indicators of these inequalities is infant mortality, which serves as a marker of social development and well-being and is a key focus in global health policies, including the United Nations’ Sustainable Development Goals and Universal Health Coverage.^6^,^8^

Extensive research has documented the impact of socioeconomic inequalities on health outcomes that exploded during the recent COVID-19 pandemic, in which there were uncovered and amplified pre-existing inequalities. Social determinants of health, such as income, education and access to healthcare, have been linked to avoidable deaths from various diseases and higher COVID-19 morbidity and mortality rates.^9 10^ These inequalities have also influenced maternal and infant health, with studies showing that disadvantaged socioeconomic conditions are associated with increased maternal mortality and disruptions in essential health services during the pandemic.

In Colombia, the relationship between social determinants and infant mortality is evident, with studies indicating that maternal education, healthcare system affiliation and residence zone significantly impact infant mortality. Recent studies highlight significant reductions in infant mortality rates (IMRs), from 33.7 per 1000 live births in 1993 to 17.3 in 2019, but also reveal that inequalities between regions, particularly about poverty, have persisted. In 1993, the difference in IMRs between the poorest (Chocó) and wealthiest (Bogotá, D.C.) departments was 63.1%, decreasing only slightly to 55.9% by 2019. Additionally, maternal education, health insurance affiliation and departmental Gross Domestic Product (GDP) play crucial roles in explaining variations in IMRs.^11 12^ A study covering 2000–2011 found that while early neonatal, late neonatal and postneonatal mortality decreased by 43%, 26% and 40%, respectively, the Amazonía and Caribe regions exhibited the highest rates. Infants born to mothers with lower education levels and those in the subsidised healthcare scheme were at significantly higher risk of mortality. Despite overall improvements, the strong association between poverty and infant mortality underscores the need for targeted policy interventions to address these enduring inequalities.^13^

Despite these findings, there is a paucity of longitudinal studies that analyse health inequalities and their relationship with social determinants over time in Colombia or other low-income and middle-income countries (LMICs). This study aims to fill this gap by quantifying the 1-year survival rates among live births in Colombia and evaluating the impact of maternal educational level, health insurance enrolment scheme and area of residence on these rates, and measuring the related socioeconomic inequalities over a decade.

## Methods

### Study design and population

This was a retrospective nationwide cohort study of all singleton live births in Colombia between 1 January 2011 and 31 December 2020. All singletons’ live births were followed up until the first year of life or death, whichever occurred first. We constructed the cohort using information from all birth certificates in the Unified Affiliation Registry (RUAF, from the Spanish name), administered by the Colombian Ministry of Health and Social Protection (MoH). Mortality information was obtained using death certificates that are in the same registry. The MoH provided the Faculty of Medicine at the Universidad Nacional de Colombia with data from all birth and death certificates registered in the RUAF for the study period. These certificates contain information about the mother (age, educational level, health system scheme of affiliation, area of residence, marital status and number of pregnancies), pregnancy details (number of prenatal check-ups, gestational age) and live birth details (sex, birth weight, APGAR scores at 1 and 5 min, birth length). RUAF contents and its operation have been assessed by international institutions, which have concluded that the system has made significant progress since its establishment in terms of coverage, completeness and timeliness. RUAF captured 99% of the births reported between 2012 and 2016 and 91% of the deaths reported in 2016 in Colombia.^14^

The final study sample included all births with complete information for all variables (n=5 605 111; 95.1% of the total database, online supplemental figure 1). We opted for a complete case analysis instead of a multiple imputation approach, as evidence indicates that a complete case analysis performs better when the proportion of missing data is not sufficiently high to compromise the validity and precision of study estimates.^15^,^17^ Differences between the included and excluded individuals are presented in online supplemental table 1.

### Patient and public involvement

Patients and the public were not involved in this research’s design, conduct, reporting or dissemination plans. The study used secondary data from the RUAF provided by the MoH and Social Protection of Colombia and, therefore, did not require direct engagement with patients or the public. The Faculty of Medicine Ethics Committee at the Universidad Nacional de Colombia approved this project (approval letter number 021-121/2020).

### Data linkage and study variables

Both birth and death certificates for infants under 1 year of age contain a maternal identifier, initially corresponding to the national identification number. That identifier was anonymised by the MoH before sharing the datasets to protect confidentiality and a different unique ID number was assigned to each mother which enabled deterministic linkage between the two datasets. We used all available birth certificates from 2011 to 2020 and death certificates from 2011 to 2021. The cohort was constructed using information from all singleton’s live births as the primary data source and under-1-year mortality certificates to determine if the live birth died before the first year of life and the date of death.

The primary outcome was 1-year survival, and three measures of socioeconomic position (SEP) were employed: maternal education, health insurance schemes and area of residence (urban municipal centre, small populated centre and dispersed rural area). Maternal education was assessed as the highest level achieved and categorised into primary school or less, secondary school, technical school or university. Three categories of health insurance were used: contributory (including exceptional and special), subsidised and uninsured. In Colombia, healthcare services are provided based on different health insurance schemes.^18^ Each family can be enrolled in one of these schemes based on the working status of their members and household resources. Based on a proxy means test, the subsidised scheme comprises those without formal employment who are classified as ‘poor’. The healthcare provided by the subsidised scheme is mainly funded through tax revenue. Those with formal employment or independent jobs pay a monthly fee in the contributory scheme. As a special form of the contributory scheme, the exceptional and special schemes include petroleum public industry workers, armed forces members, teachers in the public sector, among others. Exceptional and special schemes leave a proportion of the population (6% in our data). Finally, uninsured individuals (2 to 3% of Colombian population) are not in formal employment or able to pay a monthly fee and are not considered eligible for subsidised scheme based on the means test. Although not a direct measure of SEP, health insurance status has been used as a proxy for socioeconomic circumstances in previous analyses of health inequalities in Colombia due to the abovementioned features of the different schemes.^19 20^

Other covariates observed were the mother’s age, baby’s sex, number of previous pregnancies, mother’s marital status, prenatal check-ups during pregnancy, gestational age at birth, birth weight, birth length, APGAR score at 5 min, year of birth and geographical region of residence.

### Statistical analysis

A descriptive statistical analysis of the mothers’ sociodemographic characteristics and some perinatal outcomes was performed. Poisson distribution was used to estimate IMRs per 1000 live births and 95% CIs for the general population and each department in Colombia. Also, IMRs were estimated for each category of maternal educational level, health insurance enrolment scheme and residence zone at delivery.

HRs and 95% CIs for 1-year survival, unadjusted and adjusted, were estimated using semiparametric Cox regression models for each main exposure variable. Two adjusted models were defined. Model 1 (the main model) included maternal age, marital status, baby’s sex, year of birth and geographical region of birth. Model 2 (presented in the online supplemental material as a sensitivity analysis) additionally adjusted for number of prenatal check-ups, gestational age, birth weight, birth length and APGAR score at 5 min. The proportionality assumption was tested graphically and statistically.

To obtain results on inequalities that could be comparable between different years and with other populations and quantify the socioeconomic gradient in absolute and relative terms, we estimated the Relative Index of Inequality (RII) and the Slope Index of Inequality (SII).^21^ Following the recommendations of Moreno-Betancur *et al*, we used Poisson regression models to estimate these indices.^22^ As with the Cox models, two adjustment strategies were applied. Model 1 adjusted for maternal age and marital status, baby’s sex, year of birth and geographic region of birth. Model 2 included the full set of covariates as described above. We selected model 1 as the main specification, as it captures the effect of socioeconomic and geographic position on infant mortality, without adjusting for potential mediators in the causal pathway, that is, prenatal care and birth outcomes. Model 2 estimates are presented in the online supplemental material. Inequality indices (RII and SII) were calculated for all exposure variables: maternal educational level, health insurance enrolment scheme and residence zone at birth. These indices were estimated for the entire cohort as well as separately for each birth year, in order to explore the dynamics of socioeconomic inequalities in infant mortality over the decade.

In addition, for each year of the cohort, we estimated predicted IMRs using Poisson regression models adjusted according to model 1, stratified by categories of the main exposure variables. These predicted rates were plotted as line graphs to illustrate annual trends in 1-year mortality across socioeconomic and geographic groups throughout the study period. All analyses were conducted using Stata V.17 MP (Universidad Nacional de Colombia licence).

## Results

There were 5 951 953 live births reported in Colombia between 2011 and 2020. Of these, 5 896 517 were singleton pregnancies. Due to missing information on the variables of interest, we excluded 291 406 (4.9%) records. A total of 5 605 111 live births were included in the final cohort and followed for 1 year or until death, whichever occurred first (online supplemental figure 1). Among these live newborns, 48.7% were female, 8.4% were preterm births, and the average number of prenatal check-ups was 6.4. Maternal characteristics included 12.4% of mothers with a university education, 79.7% living in urban areas, 69.2% married and 45.1% affiliated with the contributory, special or exception health insurance enrolment scheme (table 1).

**Table 1:** Sociodemographic characteristics of the cohort of live births in Colombia, 2011–2020

	Estimates N=5605 111	
	N or mean	% or SD
Newborns’ sex—N (%)		
Male	2 877 700	51.3
Female	2 727 411	48.7
Gestational age in weeks—mean (SD)	38.4	1.8
Gestational age categories—N (%)		
Less than 37	472 242	8.4
37 or more	5 132 869	91.6
Weight at birth in grams—mean (SD)	3122.3	499.6
Weight categories—N (%)		
Less than 1500	54 333	1.0
Between 1500 and 2499	395 502	7.1
2500 or more	5 155 276	92.0
Size at birth in centimetres—mean (SD)	49.7	2.8
Size at birth categories—N (%)		
Less than 40	51 494	0.9
Between 40 and 49	2 223 466	7
50 or more	3 330 151	59.4
5 min Apgar score—mean (SD)	9.4	0.7
5 min Apgar categories—N (%)		
Less than 7	41 447	0.7
7 or more	5 563 664	99.3
Number of prenatal visits—mean (SD)	6.4	2.5
Maternal age in years—mean (SD)	25.5	6.5
Maternal age categories—N (%)		
Less than 18	577 283	10.3
Between 18 and 34	4 428 147	79.0
35 or more	599 681	10.7
Educational level—N (%)		
University	692 301	12.3
Technical	647 104	11.5
Secondary	3 419 712	61.0
Primary or less	845 994	15.2
Health insurance enrolment scheme—N (%)		
Contributory/except/special	2 528 053	45.1
Subsidised	2 911 492	51.9
Uninsured	165 566	3.0
Residence zone—N (%)		
Urban municipal centre	4 468 582	79.7
Small populated centre	399 891	7.1
Dispersed rural area	736 638	13.2
Mother’s marital state—N (%)		
Married	3 876 384	69.2
In consensual union	25 381	0.5
Divorced or widowed	792 357	14.1
Single	910 989	16.2
Number of pregnancies (including this one)		
1	2 408 496	43.0
2	1 695 443	30.2
3	836 364	14.9
4	358 466	6.4
5 or more	306 342	5.5
Geographical region of residence—N (%)		
Atlantic	1 463 811	26.1
Bogota	876 782	15.6
Central	1 278 038	22.8
Oriental	1 013 126	18.1
Pacific	805 157	14.4
Other departments	168 197	3.0
Year of birth		
2011	556 696	9.9
2012	602 463	10.7
2013	483 843	8.6
2014	592 342	10.6
2015	589 207	10.5
2016	595 597	10.6
2017	594 375	10.6
2018	562 267	10.0
2019	526 287	9.4
2020	502 034	9.0

Description of the cohort's sociodemographic characteristics.

%, Percent; N, Number; SD, Standard Deviation.

### Crude IMRs

A total of 51 973 deaths were observed, resulting in a national IMR of 9.27 per 1000 live births for the study period. The four departments with the highest IMRs were, in descending order, San Andrés and Providencia (22.01 per 1000 live births), Vaupés (16.65 per 1000 live births), Córdoba (14.20 per 1000 live births) and Chocó (13.54 per 1000 live births). The four departments with the lowest mortality rates were, in increasing order, Santander (6.54 per 1000 live births), Casanare (6.60 per 1000 live births), Boyacá (7.21 per 1000 live births) and Norte de Santander (8.46 per 1000 live births) (online supplemental figure 2).

Comparisons of crude mortality rates for each SEP category show a clear pattern of social gradients. For example, lower 1-year mortality rates were observed as maternal education increased going from 11.33 per 1000 live births among children of mothers with primary or lower education to 6.87 per 1000 live births in children of mothers with university education. Similarly, the 1-year mortality rate for children of mothers enrolled with the contributory scheme (7.79 per 1000 live births) was lower than for children of uninsured mothers (13.16 per 1000 live births) and the 1-year mortality rate of mothers living in urban municipal centres (9.10 per 1000 live births) was lower than children of mothers living in dispersed rural areas (9.96 per 1000 live births) (table 2).

**Table 2:** Unadjusted infant mortality rates (per 1000 live births) by socioeconomic status and residence zone in Colombia, 2011–2020

	Mortality rate	95% CI	P value
Educational level			
University	6.87	6.67 to 7.06	0.00
Technical	7.77	7.56 to 7.99	
Secondary	9.53	9.43 to 9.64	
Primary or less	11.33	11.11 to 11.56	
Health insurance enrolment scheme			
Contributory/except/special	7.79	7.68 to 7.90	0.00
Subsidised	10.34	10.22 to 10.46	
Uninsured	13.16	12.61 to 13.71	
Residence zone			
Urban municipal centre	9.10	9.01 to 9.19	0.00
Small populated centre	9.92	9.62 to 10.23	
Dispersed rural area	9.96	9.73 to 10.19	

Unadjusted mortality rates estimated using Poisson regressions.

CI, Confidence Interval.

### 1-year survival and inequities

**Figure 1:**
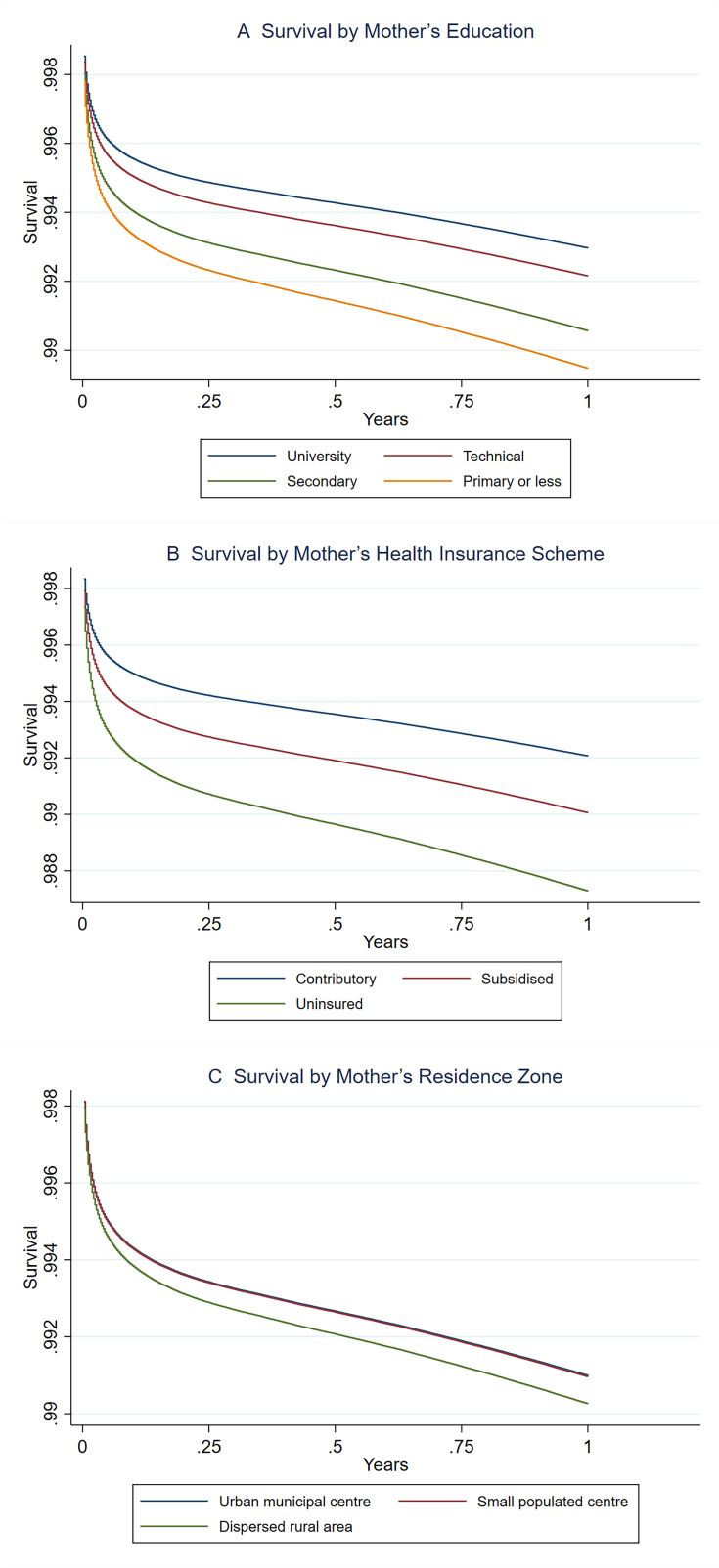
Adjusted survival curves at 1 year by maternal education, health insurance scheme and area of residence in Colombia, 2011–2020. 1-year survival using a Cox regression model controlling for mother’s age, baby’s sex, number of pregnancies, mother’s marital status, cohort year and geographical region of birth.

Table 3 presents the HRs (crude and adjusted) for different SEP categories. The risk of dying in the first year of life significantly increased as the SEP worsened, both for crude and adjusted estimates. Children of mothers with primary or lower education had a 50% higher risk of dying in the first year of life (adjusted HR (aHR) 1.50; 95% CI 1.44 to 1.56) compared with children of mothers with the highest educational level. In the same way, children of uninsured mothers had a 61% higher risk of dying in the first year of life compared with children of mothers affiliated with the contributory health scheme (aHR 1.61; 95% CI 1.54 to 1.68) (table 3). Finally, children of mothers residing in dispersed rural areas showed an 8% higher risk of dying in the first year of life compared with children of mothers living in urban municipal centres (aHR 1.08; 95% CI 1.05 to 1.11). In figure 1, survival curves from the multivariable models are shown, and a clear pattern of social gradients by education and health insurance enrolment scheme is seen (ie, the worse the mother’s socioeconomic status, the lower the observed survival). A clear difference between the dispersed rural area and the other two categories of residence zone at birth was also observed. When using model 2, which includes potential mediators such as prenatal care and birth characteristics, the magnitude of the associations was attenuated. For example, the aHR for children of mothers with primary or less education decreased from 1.50 (95% CI 1.44 to 1.56) in model 1 to 1.35 (95% CI 1.30 to 1.41) in model 2, suggesting that part of the effect of maternal education on infant mortality is mediated through these intermediate factors (see online supplemental table 2). Additionally, we estimated a joint model including the three socioeconomic dimensions simultaneously (see online supplemental table 3). The associations remained robust, suggesting that each dimension contributes independently to the risk of 1-year infant mortality. However, since we cannot infer the temporal or causal ordering between these dimensions, we prefer to present the associations from table 3 as our main results.

**Table 3:** HRs for 1-year infant mortality by socioeconomic status and residence zone in Colombia, 2011–2020

	Unadjusted HR	95% CI	P value	Adjusted HR	95% CI	P value
Educational level						
University	Reference			Reference		
Technical	1.13	1.09 to 1.18	0.00	1.12	1.07 to 1.16	0.00
Secondary	1.39	1.35 to 1.43	0.00	1.34	1.30 to 1.39	0.00
Primary or less	1.65	1.60 to 1.71	0.00	1.50	1.44 to 1.56	0.00
Health insurance enrolment scheme						
Contributory/except/special	Reference			Reference		
Subsidised	1.33	1.31 to 1.35	0.00	1.26	1.23 to 1.28	0.00
Uninsured	1.69	1.62 to 1.77	0.00	1.61	1.54 to 1.68	0.00
Residence zone						
Urban municipal centre	Reference			Reference		
Small populated centre	1.09	1.06 to 1.13	0.00	1.00	0.97 to 1.04	0.84
Dispersed rural area	1.10	1.07 to 1.12	0.00	1.08	1.05 to 1.11	0.00
Observations	5 605 111			5 605 111		

Adjusted HR using a Cox regression model controlling for mother’s age, baby’s sex, number of pregnancies, mother’s marital status, cohort year and geographical region of birth.

CI, Confidence Interval; HR, Hazard Ratio.

**Figure 2:**
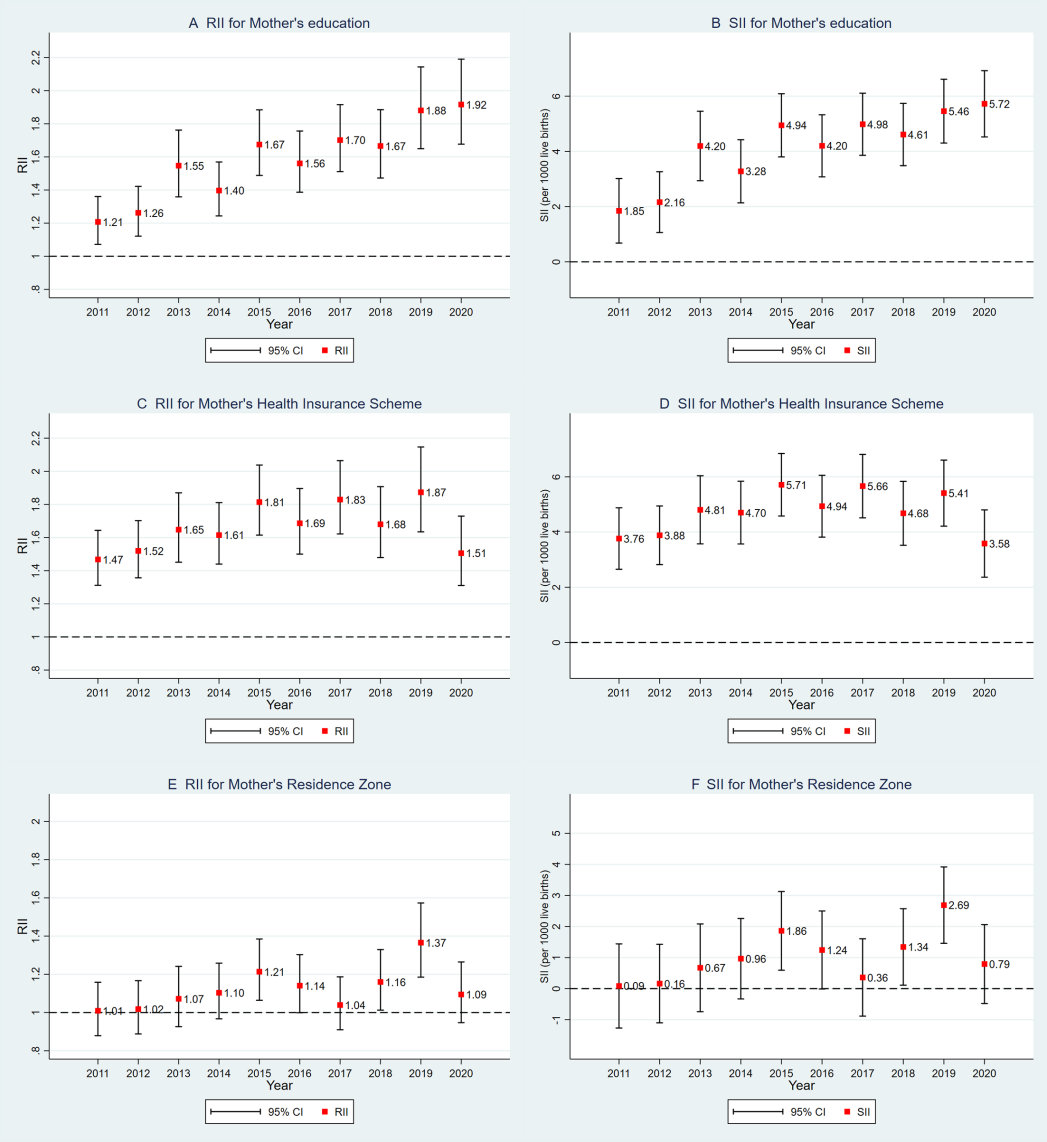
Trends in relative and absolute inequalities in infant mortality by maternal education, health insurance scheme and area of residence in Colombia, 2011–2020. Inequalities indexes estimated from a Poisson model controlling for mother’s age, baby’s sex, number of pregnancies, mother’s marital status, cohort year and geographical region of birth. RII, Relative Index of Inequality. SII, Slope Index of Inequality.

Results from the RII and SII showed persistent social gradients in infant mortality across all three dimensions of SEP: maternal education, health system enrolment scheme and area of residence (figure 2 and online supplemental table 4). Educational inequalities exhibited the most pronounced upward trend throughout the study period. Between 2011 and 2020, the RII for maternal education increased from 1.21 to 1.92, and the SII from 1.85 to 5.72, indicating a substantial and widening gap in infant mortality between the highest and lowest educational strata. Inequalities related to the health insurance enrolment scheme fluctuated over time, with the SII ranging from 3.76 in 2011, peaking at 5.71 in 2015, and declining to 3.58 in 2020. The RII for this dimension showed relatively minor variation, remaining around 1.5 throughout the decade. In contrast, inequalities by area of residence were less consistent; the RII and SII estimates fluctuated over the years and were frequently non-significant, suggesting weaker and less stable patterns of geographical disparity in infant mortality. Results from model 2, which included additional perinatal and birth-related variables, generally showed lower RII and SII values across maternal education and health insurance enrolment scheme compared with model 1 (online supplemental tables 5 and 6). This attenuation suggests that part of the association between the studied social determinants and infant mortality may be mediated through intermediate factors such as prenatal care and birth conditions. However, for area of residence (online supplemental table 7), no consistent reduction in inequality indices was observed under model 2, which may reflect the weaker or more complex role of geographic factors in the causal pathway.

Predicted IMRs estimated from model 1 for each exposure category and year are shown in online supplemental figure 3. A clear and consistent decline in mortality rates was observed among children born to mothers with university or technical education, whereas mortality rates among children of mothers with no or primary education remained relatively constant or even increased throughout the decade. This divergence explains the widening educational inequalities over time. For the health system enrolment scheme, both the contributory and subsidised groups experienced a decrease in predicted mortality, although the decline was greater in the contributory scheme. In contrast, the uninsured population showed no consistent improvement in mortality rates over the study period. These patterns suggest that improvements in infant survival have not been equitably distributed across social groups.

## Discussion

This study used administrative national data from an entire decade to identify more than 5.6 million single live births, determine IMRs and assess their association with maternal educational level, health system enrolment scheme and residence zone at birth. The results highlight significant inequalities in infant mortality across all three socioeconomic and demographic indicators. Additionally, this study found that these inequalities increased over the study period, at least in terms of the educational dimension. Although these findings were consistent with other studies demonstrating socioeconomic inequalities in childhood health outcomes in high-income and LMICs, this is the first population-based study that allows estimating the 1-year mortality risk and associating it with the mother’s socioeconomic conditions for all live births over a decade in an LMIC.

Our analyses revealed substantial inequalities in the 1-year mortality risk among children in Colombia, stratified by maternal educational level. Specifically, children of mothers with university education exhibited significantly lower 1-year mortality rates compared with those whose mothers had only primary education or less, secondary education or technical education. Furthermore, children of mothers who were either not affiliated with the health system or affiliated with the subsidised scheme showed higher mortality rates than those whose mothers were affiliated with the contributory scheme. These socioeconomic factors likely influenced 1-year survival through various pathways, including pregnancy characteristics and perinatal outcomes.^23^ This study’s findings persisted in multivariate models, even after adjusting for prenatal care and birth characteristics, such as birth weight, birth length, gestational age and the 5 min APGAR score. Notably, when comparing estimates from two models—with and without adjustment for variables of prenatal care and birth outcomes—the observed reduction in effect sizes suggests that part of the association between SEP and infant mortality may be mediated through intermediate factors related to access to and quality of prenatal care, and that another part of the causal mechanism operates through pathways beyond those directly related to pregnancy and immediate perinatal outcomes.

The trends observed in the RII and SII over the study period indicate a widening gap in infant mortality based on maternal education. The increasing trend in inequalities related to educational attainment is particularly concerning. Although the trend for health system affiliation is not evident, the lack of progress in this area is also troubling. While overall mortality declined over the decade, this improvement was not equitably distributed. The most substantial reductions were observed among children of mothers with higher socioeconomic status—particularly those with university or technical education—and those enrolled in contributory or subsidised health insurance schemes. In contrast, children of mothers with no or only primary education, as well as those who were uninsured, experienced worsening, stagnant or only marginal improvements in mortality rates, with some years showing slight increases. Similar disparities were evident by area of residence: the most consistent and marked improvements occurred among women living in urban municipal centres, while more modest, yet positive, trends were observed in dispersed rural areas—with the exception of a reversal in 2020. These findings suggest that, despite overall national progress, social and geographic inequalities in infant survival have widened due to uneven gains across population subgroups.

Despite Colombia’s economic growth, social policies have yet to effectively reach the most vulnerable populations, leading to persistent or increasing inequalities.^24^ Additionally, although the current Colombian health system has undergone reforms aimed at reducing the gaps between contributory and subsidised schemes and has been characterised by increased health system affiliation coverage and low out-of-pocket expenses,^25^ it has not succeeded in providing adequate access to health services for the entire population, especially those most in need.^26^ This suggests that social policies, including those related to the health system, must be re-evaluated and redesigned to have a tangible impact on vulnerable populations.

This study has several strengths. The first is that it enabled the deterministic linkage of birth certificate information with death certificates using an anonymised ID for the mother. This allowed for the estimation of the risk of death for each single live birth over the past decade in Colombia. Additionally, it enabled the identification of characteristics of the live birth at the time of birth and, therefore, the estimation of the association between 1-year survival and socioeconomic conditions or health system affiliation. Most studies that have estimated socioeconomic inequalities in infant mortality have used demographic and health surveys, observing a sample of the entire population.^27^,^31^ As previously mentioned, birth and death certificates in Colombia have been evaluated by different institutions and have shown good quality and virtually full population coverage. Another strength of our study is the ability to estimate 1-year survival. Most studies assessing infant mortality used cumulative mortality incidence rather than time-to-event measures, thereby losing information related to the dynamics of the risk of death. Finally, the study explored three different dimensions of inequality, using robust methodologies that allowed for comparing inequalities across years in relative and absolute terms.

However, our study has some limitations. First, we used birth and death certificates from the RUAF, managed by the MoH, rather than those from the National Administrative Department of Statistics (DANE), the central public agency for managing vital statistics. Although RUAF is the primary source for over 90% of the birth and death certificates used by DANE, the latter complements this data with additional sources. Nevertheless, DANE does not authorise access to microdata with individual identification. Second, we did not include all live births, as we excluded multiple pregnancies or live birth records lacking complete information. While these decisions improved the internal validity of our results, they also reduced the generalisability of the findings and resulted in a population with lower 1-year mortality rates than those reported by official sources for Colombia (the World Bank’s observatory shows that in 2019, Colombia had an IMR of around 11 per 1000 live births).^32^ The observed differences in sociodemographic characteristics between included and excluded individuals, that is, excluded mothers had poorer socioeconomic conditions than those included, suggest a potential selection bias, possibly leading to an underestimation of the true socioeconomic inequalities in infant mortality. Third, although we adjusted for all observable variables we considered relevant, other unobservable variables may not be included in the models, which could lead to residual confounding. Fourth, health insurance status was recorded at birth, and potential changes during the first year of life were not captured. However, Colombian law guarantees continuous insurance coverage under both contributory and subsidised schemes throughout the child’s first year of life, regardless of any changes in parental employment status. Lastly, although the COVID-19 pandemic may have disproportionately affected vulnerable populations, the first wave in Colombia began in July 2020 and the second in January 2021; thus, its potential effect would be limited to births from the last semester of our study period and is unlikely to have substantially influenced our results.

In conclusion, our findings underscore the critical need for public policies targeting the known structural determinants of child health together with specific interventions to reduce socioeconomic and healthcare inequalities in infant mortality in Colombia. Efforts to improve maternal education, the health system and enhance social conditions in vulnerable groups are essential to reducing infant mortality and promoting equitable health outcomes for all children. Future research should explore the underlying mechanisms driving these disparities and evaluate the effectiveness of specific policy interventions in addressing them.

## Supplementary material

10.1136/bmjgh-2024-018526 online supplemental file 1

## Data Availability

Data may be obtained from a third party and are not publicly available.
