# Supplementary material for: Socioeconomic inequalities in infant mortality in Colombia: a nationwide cohort study during 10 years
Source: BMJ Glob Health. 2025 Aug 21;10(8):e018526. doi: 10.1136/bmjgh-2024-018526 (PMC12374676; doi:10.1136/bmjgh-2024-018526)
Supplement: online supplemental file 1 [file bmjgh-10-8-s001.docx]

**Supplemental Material**

**Socioeconomic inequalities in infant mortality in Colombia: A Nation-Wide Cohort Study during 10 years**

**Content**

[A.1. Description of sources and quality of information 2](#_Toc202911367)

[Supplemental Table 1. Comparison between included and excluded live births in Colombia, 2011-2020. 3](#_Toc202911368)

[Supplemental Table 2. Hazard ratios (HR) for 1-year infant mortality by socioeconomic position and residence zone in Colombia, 2011-2020. Model 2. 6](#_Toc202911369)

[Supplemental Table 3. Hazard ratios (HR) for 1-year infant mortality by socioeconomic position and residence zone in Colombia, 2011-2020. Joint Model. 7](#_Toc202911370)

[Supplemental Table 4. Relative Index of Inequality (RII) and Slope Index of Inequality (SII) for socioeconomic inequalities in infant mortality in Colombia, 2011-2020. Model 1. 8](#_Toc202911371)

[Supplemental Table 5. Relative Index of Inequality (RII) and Slope Index of Inequality (SII) for maternal educational level among live births in Colombia, 2011-2020. Models 1 and 2. 9](#_Toc202911372)

[Supplemental Table 6. Relative Index of Inequality (RII) and Slope Index of Inequality (SII) for maternal health insurance scheme among live births in Colombia, 2011-2020. Models 1 and 2. 10](#_Toc202911373)

[Supplemental Table 7. Relative Index of Inequality (RII) and Slope Index of Inequality (SII) for maternal residence zone among live births in Colombia, 2011-2020. Models 1 and 2. 11](#_Toc202911374)

[Supplemental Figure 1. Sources of information and study population 12](#_Toc202911375)

[Supplemental Figure 2. Geographical distribution of infant mortality rate in Colombia by departments 13](#_Toc202911376)

[Supplemental Figure 3. Trends in predicted infant mortality rates by maternal education, health insurance scheme, and area of residence in Colombia, 2011–2020 14](#_Toc202911377)

# **A.1. Description of sources and quality of information**

Our study included singleton live births between January 1st, 2011, and December 31st, 2020, and those followed up until the first year of life or death, whichever occurred first. To identify this population, we used the following database:

1. Our primary source of information was the **Single Registry of Enrollees, Module ND** (RUAF, from Spanish name), administered by the Ministry of Health and Social Protection. This is the main source that the National Administrative Department of Statistics (*Departamento Administrativo Nacional de Estadística,* or DANE) uses to generate the country's vital statistics. RUAF was created in 2007 through the *Circular Externa Conjunta* No. 0081 of November 13th, 2007. RUAF contents and its operation have been assessed by international institutions, which have concluded that the system has made great progress since its establishment in terms of coverage, completeness, and timeliness.^[[1]](#footnote-1)^ 99% of the births reported in Colombia between 2012 and 2016 were reported in the ND module; for 2016, 91% of the deaths were reported in the ND module. The main reason for the latter gap was that not all deaths verified by the National Institute of Legal Medicine and Forensic Sciences were registered in RUAF’s Module ND (these deaths are related to external causes, namely homicides and traffic accidents).^[[2]](#footnote-2)^

# **Supplemental Table 1. Comparison between included and excluded live births in Colombia, 2011-2020.**

|  | Included N=5,605,111 | | Excluded N=291,406 | | p-value | SMD/SPD |
| --- | --- | --- | --- | --- | --- | --- |
|  | N or Mean or Median | % or SD or IQR | N or Mean or Median | % or SD or IQR |  |  |
|  |  |  |  |  |  |  |
| New-born's sex - N (%) |  |  |  |  | 0.32 | 0.00 |
| Male | 2,877,700 | 51.34 | 149,152 | 51.25 |  |  |
| Female | 2,727,411 | 48.66 | 141,899 | 48.75 |  |  |
| Gestational age in weeks |  |  |  |  | < 0.001 | 0.37 |
| Mean (SD) | 38.4 | 1.8 | 38.31 | 1.9 |  |  |
| Median (IQR) | 39 | 1 | 39 | 1 |  |  |
| Gestational age categories - N (%) |  |  |  |  | 0.73 | 0.00 |
| Less than 37 | 472,242 | 8.43 | 24,605 | 8.44 |  |  |
| 37 or more | 5,132,869 | 91.57 | 266,801 | 91.56 |  |  |
| Weight at birth in grams |  |  |  |  | < 0.001 | 0.03 |
| Mean (SD) | 3,122.33 | 499.59 | 3,105.41 | 525.45 |  |  |
| Median (IQR) | 3,145 | 578 | 3,100 | 615 |  |  |
| Weight categories - N (%) |  |  |  |  | < 0.001 | 0.02 |
| Less than 1500 | 54,333 | 0.97 | 3,466 | 1.19 |  |  |
| Between 1500 and 2499 | 395,502 | 7.06 | 19,835 | 6.81 |  |  |
| 2500 or more | 5,155,276 | 91.97 | 268,105 | 92 |  |  |
| Size at birth in centimetres |  |  |  |  | < 0.001 | 0.07 |
| Mean (SD) | 49.72 | 2.84 | 49.5 | 3.22 |  |  |
| Median (IQR) | 50 | 3 | 50 | 3 |  |  |
| Size at birth categories - N (%) |  |  |  |  | < 0.001 | 0.04 |
| Less than 40 | 51,494 | 0.92 | 3,918 | 1.34 |  |  |
| Between 40 and 49 | 2,223,466 | 39.67 | 113,837 | 39.06 |  |  |
| 50 or more | 3,330,151 | 59.41 | 173,651 | 59.59 |  |  |
| Five-minute Apgar score |  |  |  |  | < 0.001 | 0.07 |
| Mean (SD) | 9.42 | 0.75 | 9.44 | 0.89 |  |  |
| Median (IQR) | 10 | 1 | 10 | 1 |  |  |
| Five-minute Apgar categories - N (%) |  |  |  |  | < 0.001 | 0.04 |
| 7 or more | 41,447 | 0.74 | 3,315 | 1.14 |  |  |
| Less than 7 | 5,563,664 | 99.26 | 288,091 | 98.86 |  |  |
| Number of prenatal visits - Mean (SD) |  |  |  |  | < 0.001 | 0.53 |
| Mean (SD) | 6.42 | 2.53 | 5.03 | 2.69 |  |  |
| Median (IQR) | 7 | 3 | 5 | 4 |  |  |
| Maternal age in years - Mean (SD) |  |  |  |  | < 0.001 |  |
| Mean (SD) | 25.49 | 6.53 | 25.32 | 7.37 |  |  |
| Median (IQR) | 25 | 10 | 24 | 10 |  |  |
| Maternal age categories - N (%) |  |  |  |  | < 0.001 | 0.07 |
| Less than 18 | 577,283 | 10.30 | 34,852 | 11.96 |  |  |
| Between 18 and 34 | 4,428,147 | 79.00 | 222,357 | 76.3 |  |  |
| 35 or more | 599,681 | 10.70 | 34,197 | 11.74 |  |  |
| Educational level - N (%) |  |  |  |  | < 0.001 | 0.84 |
| University | 692,301 | 12.35 | 2,692 | 3.02 |  |  |
| Technical | 647,104 | 11.54 | 2,419 | 2.71 |  |  |
| Secondary | 3,419,712 | 61.01 | 41,740 | 46.82 |  |  |
| Primary or less | 845,994 | 15.09 | 42,303 | 47.45 |  |  |
| Social security scheme - N (%) |  |  |  |  | < 0.001 | 0.57 |
| Contributory/Except/Special | 2,528,053 | 45.10 | 57,389 | 19.69 |  |  |
| Subsidised | 2,911,492 | 51.94 | 217,258 | 74.56 |  |  |
| Uninsured | 165,566 | 2.95 | 16,758 | 5.75 |  |  |
| Residence zone - N (%) |  |  |  |  | < 0.001 | 0.33 |
| Urban municipal centre | 4,468,582 | 79.72 | 189,068 | 65.37 |  |  |
| Small populated centre | 399,891 | 7.13 | 25,700 | 8.89 |  |  |
| Dispersed rural area | 736,638 | 13,14 | 74,478 | 13,76 |  |  |
| Mother's marital state - N (%) |  |  |  |  | < 0.001 | 0.17 |
| Married | 3,876,384 | 69.16 | 18,096 | 10.97 |  |  |
| In consensual union | 25,381 | 0.45 | 124,537 | 75.52 |  |  |
| Divorced or widowed | 792,357 | 14.14 | 956 | 0.58 |  |  |
| Single | 910,989 | 16.25 | 21,307 | 12.92 |  |  |
| Number of pregnancies (including this one) |  |  |  |  | < 0.001 | 0.25 |
| One | 2,408,496 | 42.97 | 137,377 | 47.14 |  |  |
| Two | 1,695,443 | 30.25 | 64,654 | 22.19 |  |  |
| Three | 836,364 | 14.92 | 38,862 | 13.34 |  |  |
| Four | 358,466 | 6.40 | 20,845 | 7.15 |  |  |
| Five or more | 306,342 | 5.47 | 29,668 | 10.18 |  |  |
| Geographical region of residence - N (%) |  |  |  |  | < 0.001 | 0.52 |
| Atlantic | 1,463,811 | 26.12 | 128,606 | 44.46 |  |  |
| Bogota | 876,782 | 15.64 | 15,938 | 5.51 |  |  |
| Central | 1,278,038 | 22.80 | 48,317 | 16.7 |  |  |
| Oriental | 1,013,126 | 18.08 | 33,774 | 11.68 |  |  |
| Pacific | 805,157 | 14.36 | 46,229 | 15.98 |  |  |
| Other departments | 168,197 | 3.00 | 16,382 | 5.66 |  |  |
| Year of birth |  |  |  |  | < 0.001 | 0.11 |
| 2011 | 556,696 | 9.93 | 23,158 | 7.95 |  |  |
| 2012 | 602,463 | 10.75 | 28,139 | 9.66 |  |  |
| 2013 | 483,843 | 8.63 | 26,315 | 9.03 |  |  |
| 2014 | 592,342 | 10.57 | 28,280 | 9.7 |  |  |
| 2015 | 589,207 | 10.51 | 30,560 | 10.49 |  |  |
| 2016 | 595,597 | 10.63 | 31,990 | 10.98 |  |  |
| 2017 | 594,375 | 10.60 | 32,252 | 11.07 |  |  |
| 2018 | 562,267 | 10.03 | 35,334 | 12.13 |  |  |
| 2019 | 526,287 | 9.39 | 29,962 | 10.28 |  |  |
| 2020 | 502,034 | 8.96 | 25,416 | 8.72 |  |  |

*Comparison between individuals included and excluded from the analysis due to missing values. The distribution of variables was compared using Chi-square tests for categorical variables and Kruskal-Wallis tests for continuous variables (none of the continuous variables met normality criteria). Additionally, to account for significant differences due to sample size, standardized mean differences (SMD) for continuous variables and standardized proportion differences (SPD) for categorical variables were estimated. An SMD/SPD < 0.1 indicates good balance.*

*SD: Standard Deviation; IQR: Interquartile Range (75th percentile - 25th percentile).*

# **Supplemental Table 2. Hazard ratios (HR) for 1-year infant mortality by socioeconomic position and residence zone in Colombia, 2011-2020. Model 2.**

|  |  | Adjusted HR | 95% CI | | | p-value |
| --- | --- | --- | --- | --- | --- | --- |
|  |  |  |  |  |  |  |
| Educational level |  |  |  |  |  |  |
| University |  | Reference |  |  |  |  |
| Technical |  | 1.05 | 1.01 | to | 1.10 | 0.00 |
| Secondary |  | 1.19 | 1.15 | to | 1.23 | 0.00 |
| Primary or less |  | 1.35 | 1.30 | to | 1.41 | 0.00 |
|  |  |  |  |  |  |  |
| Social security scheme |  |  |  |  |  |  |
| Contributory/Except/Special |  | Reference |  |  |  |  |
| Subsidised |  | 1.21 | 1.19 | to | 1.24 | 0.00 |
| Uninsured |  | 1.22 | 1.17 | to | 1.27 | 0.00 |
|  |  |  |  |  |  |  |
| Residence zone |  |  |  |  |  |  |
| Urban municipal centre |  | Reference |  |  |  |  |
| Small populated centre |  | 1.04 | 1.01 | to | 1.04 | 0.00 |
| Dispersed rural area |  | 1.12 | 1.08 | to | 1.11 | 0.00 |
|  |  |  |  |  |  |  |
| Observations |  | 5,605,111 |  |  |  |  |

Model 2: Cox regression models adjusted by mother's age, baby's sex, number of pregnancies, mother's marital status, number of prenatal check-ups, gestational age, birth weight, birth length, 5-minute APGAR score, year of birth, and geographical region of birth.

# **Supplemental Table 3. Hazard ratios (HR) for 1-year infant mortality by socioeconomic position and residence zone in Colombia, 2011-2020. Joint Model.**

|  |  | Adjusted HR | 95% CI | | | p-value |
| --- | --- | --- | --- | --- | --- | --- |
|  |  |  |  |  |  |  |
| Educational level |  |  |  |  |  |  |
| University |  | Reference |  |  |  |  |
| Technical |  | 1.10 | 1.06 | to | 1.15 | 0.00 |
| Secondary |  | 1.25 | 1.21 | to | 1.30 | 0.00 |
| Primary or less |  | 1.36 | 1.30 | to | 1.41 | 0.00 |
|  |  |  |  |  |  |  |
| Social security scheme |  |  |  |  |  |  |
| Contributory/Except/Special |  | Reference |  |  |  |  |
| Subsidised |  | 1.18 | 1.16 | to | 1.21 | 0.00 |
| Uninsured |  | 1.53 | 1.45 | to | 1.60 | 0.00 |
|  |  |  |  |  |  |  |
| Residence zone |  |  |  |  |  |  |
| Urban municipal centre |  | Reference |  |  |  |  |
| Small populated centre |  | 0.94 | 0.91 | to | 0.97 | 0.00 |
| Dispersed rural area |  | 1.00 | 0.97 | to | 1.02 | 0.90 |
|  |  |  |  |  |  |  |
| Observations |  | 5,605,111 |  |  |  |  |

Model: Cox regression model adjusted by mother's educational level, mother's social security scheme, mother's residence zone, mother's age, baby's sex, number of pregnancies, mother's marital status, number of prenatal check-ups, gestational age, birth weight, birth length, 5-minute APGAR score, year of birth, and geographical region of birth.

# **Supplemental Table 4. Relative Index of Inequality (RII) and Slope Index of Inequality (SII) for socioeconomic inequalities in infant mortality in Colombia, 2011-2020. Model 1.**

|  | Index | 95% CI | | | p-value |
| --- | --- | --- | --- | --- | --- |
|  |  |  |  |  |  |
| Educational level |  |  |  |  |  |
| RII | 1.55 | 1.49 | to | 1.62 | 0.00 |
| SII | 4.12 | 3.76 | to | 4.48 | 0.00 |
|  |  |  |  |  |  |
| Health insurance scheme |  |  |  |  |  |
| RII | 1.67 | 1.60 | to | 1.73 | 0.00 |
| SII | 4.78 | 4.42 | to | 5.14 | 0.00 |
|  |  |  |  |  |  |
| Residence zone |  |  |  |  |  |
| RII | 1.12 | 1.07 | to | 1.17 | 0.00 |
| SII | 1.04 | 0.63 | to | 1.44 | 0.00 |

*Legend: RII: Relative Index of Inequality; SII: Slope Index of Inequality. Poisson regression models adjusted by maternal age and marital status, baby's sex, year of birth, and geographical region of birth.*

# **Supplemental Table 5. Relative Index of Inequality (RII) and Slope Index of Inequality (SII) for maternal educational level among live births in Colombia, 2011-2020. Models 1 and 2.**

| **Year** | **Index** | **Model 1** | **Model 2** |
| --- | --- | --- | --- |
| 2011 | RII | 1.21 (1.07–1.36) | 1.18 (1.05–1.34) |
|  | SII | 1.85 (0.68–3.02) | 2.58 (0.71–4.45) |
| 2012 | RII | 1.26 (1.12–1.42) | 1.22 (1.08–1.38) |
|  | SII | 2.16 (1.06–3.26) | 3.31 (1.24–5.38) |
| 2013 | RII | 1.55 (1.36–1.76) | 1.38 (1.21–1.58) |
|  | SII | 4.20 (2.94–5.45) | 4.63 (2.74–6.52) |
| 2014 | RII | 1.40 (1.24–1.57) | 1.38 (1.23–1.55) |
|  | SII | 3.28 (2.14–4.42) | 5.19 (3.29–7.09) |
| 2015 | RII | 1.67 (1.49–1.88) | 1.56 (1.39–1.76) |
|  | SII | 4.94 (3.80–6.09) | 7.05 (5.14–8.95) |
| 2016 | RII | 1.56 (1.39–1.76) | 1.30 (1.15–1.46) |
|  | SII | 4.20 (3.08–5.33) | 4.38 (2.35–6.41) |
| 2017 | RII | 1.70 (1.51–1.92) | 1.66 (1.47–1.87) |
|  | SII | 4.98 (3.86–6.11) | 8.25 (6.24–10.27) |
| 2018 | RII | 1.67 (1.47–1.89) | 1.40 (1.23–1.58) |
|  | SII | 4.61 (3.48–5.74) | 6.72 (4.20–9.23) |
| 2019 | RII | 1.88 (1.65–2.14) | 1.78 (1.56–2.04) |
|  | SII | 5.46 (4.30–6.61) | 9.09 (6.92–11.26) |
| 2020 | RII | 1.92 (1.68–2.19) | 1.53 (1.33–1.75) |
|  | SII | 5.72 (4.52–6.92) | 7.28 (4.92–9.64) |
| **Total** | **RII** | **1.55 (1.49–1.62)** | **1.43 (1.38–1.49)** |
|  | **SII** | **4.12 (3.76–4.48)** | **5.71 (5.08–6.33)** |

*Model 1: Poisson regression models adjusted by maternal age and marital status, baby's sex, year of birth, and geographical region of birth.*

*Model 2: Poisson regression models adjusted by mother's age, baby's sex, number of pregnancies, mother's marital status, number of prenatal check-ups, gestational age, birth weight, birth length, 5-minute APGAR score, year of birth, and geographical region of birth.*

# **Supplemental Table 6. Relative Index of Inequality (RII) and Slope Index of Inequality (SII) for maternal health insurance scheme among live births in Colombia, 2011-2020. Models 1 and 2.**

| **Year** | **Index** | **Model 1** | **Model 2** |
| --- | --- | --- | --- |
| 2011 | RII | 1.47 (1.31–1.64) | 1.28 (1.14–1.43) |
|  | SII | 3.76 (2.65–4.88) | 3.74 (1.97–5.51) |
| 2012 | RII | 1.52 (1.36–1.70) | 1.31 (1.17–1.47) |
|  | SII | 3.88 (2.82–4.94) | 4.58 (2.64–6.52) |
| 2013 | RII | 1.65 (1.45–1.87) | 1.47 (1.29–1.67) |
|  | SII | 4.81 (3.57–6.04) | 5.49 (3.62–7.37) |
| 2014 | RII | 1.61 (1.44–1.81) | 1.34 (1.19–1.51) |
|  | SII | 4.70 (3.57–5.84) | 4.71 (2.83–6.59) |
| 2015 | RII | 1.81 (1.61–2.04) | 1.28 (1.14–1.44) |
|  | SII | 5.71 (4.58–6.85) | 3.87 (2.02–5.71) |
| 2016 | RII | 1.69 (1.50–1.90) | 1.43 (1.27–1.61) |
|  | SII | 4.94 (3.82–6.05) | 6.01 (3.97–8.05) |
| 2017 | RII | 1.83 (1.62–2.06) | 1.62 (1.43–1.83) |
|  | SII | 5.66 (4.51–6.81) | 7.77 (5.79–9.75) |
| 2018 | RII | 1.68 (1.48–1.91) | 1.47 (1.30–1.67) |
|  | SII | 4.68 (3.52–5.84) | 7.77 (5.21–10.33) |
| 2019 | RII | 1.87 (1.63–2.15) | 2.08 (1.81–2.39) |
|  | SII | 5.41 (4.21–6.60) | 11.63 (9.33–13.93) |
| 2020 | RII | 1.51 (1.31–1.73) | 1.25 (1.09–1.44) |
|  | SII | 3.58 (2.36–4.80) | 3.86 (1.43–6.29) |
| **Total** | **RII** | **1.67 (1.60–1.73)** | **1.43 (1.37–1.48)** |
|  | **SII** | **4.78 (4.42–5.14)** | **5.60 (4.98–6.21)** |

*Model 1: Poisson regression models adjusted by maternal age and marital status, baby's sex, year of birth, and geographical region of birth.*

*Model 2: Poisson regression models adjusted by mother's age, baby's sex, number of pregnancies, mother's marital status, number of prenatal check-ups, gestational age, birth weight, birth length, 5-minute APGAR score, year of birth, and geographical region of birth.*

# **Supplemental Table 7. Relative Index of Inequality (RII) and Slope Index of Inequality (SII) for maternal residence zone among live births in Colombia, 2011-2020. Models 1 and 2.**

| **Year** | **Index** | **Model 1** | **Model 2** |
| --- | --- | --- | --- |
| 2011 | RII | 1.01 (0.88–1.16) | 1.20 (1.04–1.37) |
|  | SII | 0.09 (-1.27–1.44) | 2.76 (0.61–4.91) |
| 2012 | RII | 1.02 (0.89–1.17) | 1.05 (0.92–1.21) |
|  | SII | 0.16 (-1.10–1.43) | 0.85 (-1.47–3.16) |
| 2013 | RII | 1.07 (0.93–1.24) | 1.24 (1.07–1.43) |
|  | SII | 0.67 (-0.74–2.08) | 3.07 (0.93–5.20) |
| 2014 | RII | 1.10 (0.97–1.26) | 1.18 (1.03–1.34) |
|  | SII | 0.96 (-0.33–2.26) | 2.61 (0.47–4.75) |
| 2015 | RII | 1.21 (1.06–1.38) | 1.34 (1.17–1.53) |
|  | SII | 1.86 (0.59–3.13) | 4.65 (2.53–6.78) |
| 2016 | RII | 1.14 (1.00–1.30) | 1.25 (1.09–1.43) |
|  | SII | 1.24 (-0.01–2.50) | 3.77 (1.49–6.05) |
| 2017 | RII | 1.04 (0.91–1.19) | 1.13 (0.99–1.30) |
|  | SII | 0.36 (-0.89–1.60) | 2.03 (-0.16–4.21) |
| 2018 | RII | 1.16 (1.01–1.33) | 1.33 (1.16–1.52) |
|  | SII | 1.34 (0.11–2.57) | 5.78 (2.94–8.61) |
| 2019 | RII | 1.37 (1.19–1.57) | 1.45 (1.26–1.67) |
|  | SII | 2.69 (1.46–3.92) | 5.83 (3.57–8.09) |
| 2020 | RII | 1.09 (0.95–1.26) | 0.92 (0.79–1.06) |
|  | SII | 0.79 (-0.48–2.06) | -1.52 (-4.04–1.00) |
| **Total** | **RII** | **1.12 (1.07–1.17)** | **1.21 (1.16–1.26)** |
|  | **SII** | **1.04 (0.63–1.44)** | **3.02 (2.33–3.72)** |

*Model 1: Poisson regression models adjusted by maternal age and marital status, baby's sex, year of birth, and geographical region of birth.*

*Model 2: Poisson regression models adjusted by mother's age, baby's sex, number of pregnancies, mother's marital status, number of prenatal check-ups, gestational age, birth weight, birth length, 5-minute APGAR score, year of birth, and geographical region of birth.*

# **Supplemental Figure 1. Sources of information and study population**

# **Supplemental Figure 2. Geographical distribution of infant mortality rate in Colombia by departments**


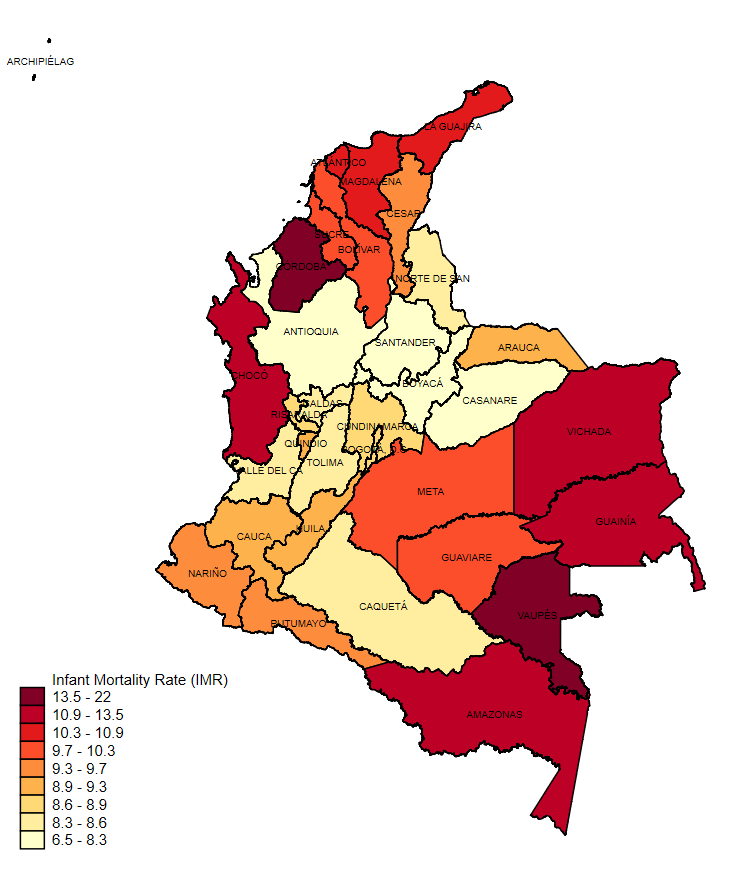


# **Supplemental Figure 3. Trends in predicted infant mortality rates by maternal education, health insurance scheme, and area of residence in Colombia, 2011–2020**

A. Mortality rates by maternal Education Level


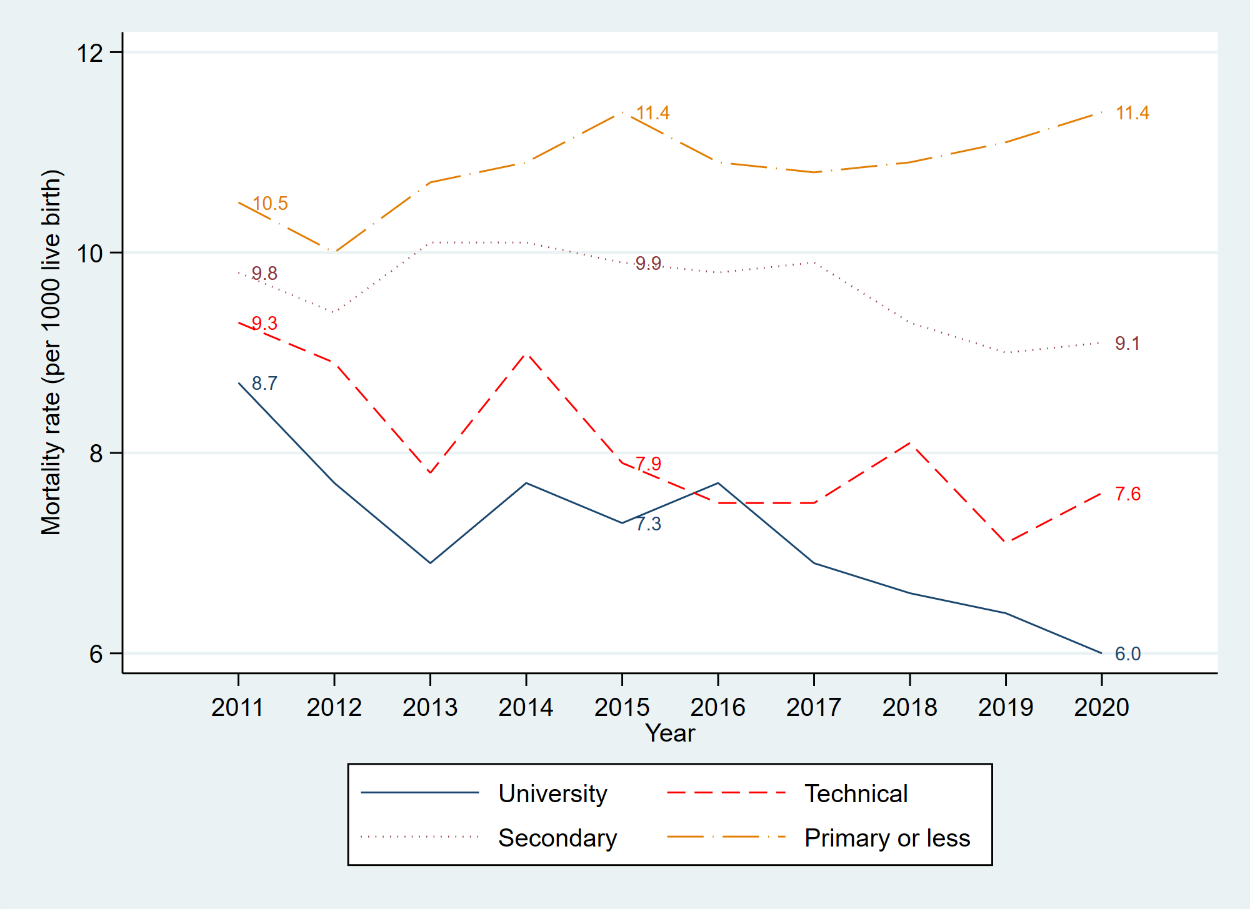


B. Mortality rates by maternal Health Insurance Scheme


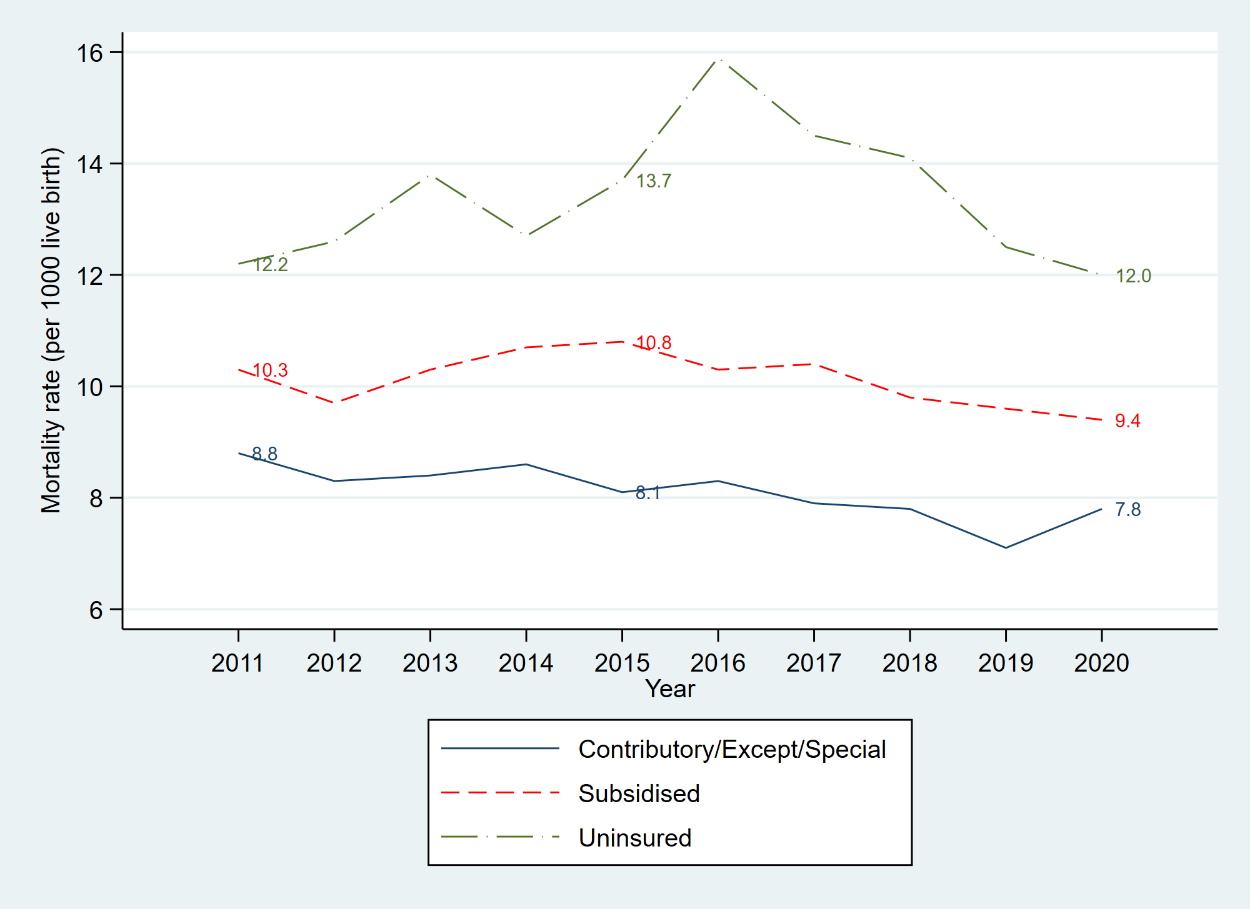


C. Mortality rates by maternal Residence Zone


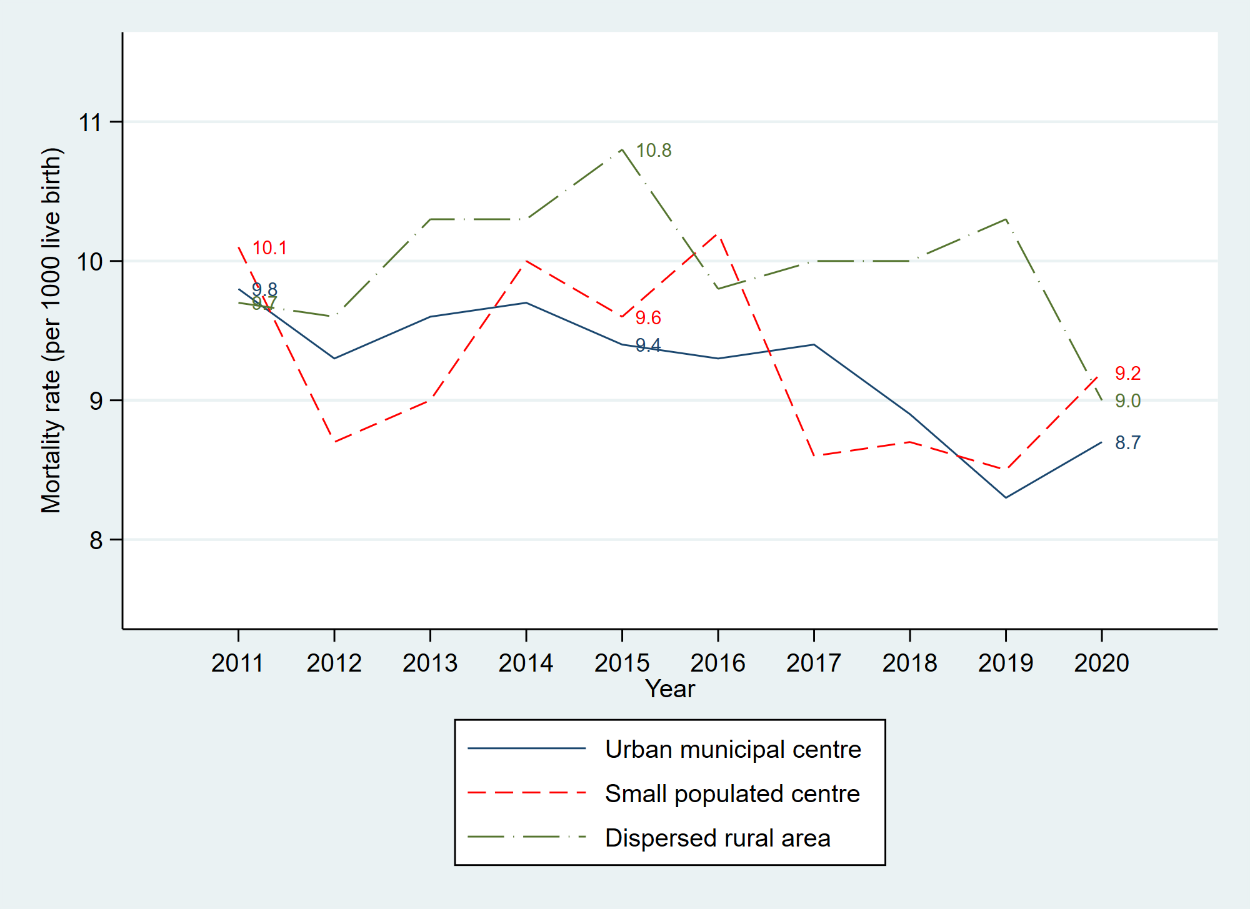


*Predicted rates using Poisson regression models adjusted by maternal age and marital status, baby's sex, year of birth, and geographical region of birth.*

1. Colombia Implementation Working Group. Colombia: A strategy to improve the registration and certification of vital events in rural and ethnic communities. CRVS country perspectives. Melbourne, Australia: Bloomberg Philanthropies Data for Health Initiative, Civil Registration and Vital Statistics Improvement, the University of Melbourne; 2018 [↑](#footnote-ref-1)
2. Toro Roa, Juan Pablo; Iunes, Roberto F.; Mills, Samuel. 2019. Achieving Health Outcomes in Colombia: Civil Registration and Vital Statistics System, Unique Personal Identification Number, and Unified Beneficiary Registry System for Births and Deaths. Health, Nutrition, and Population Discussion Paper; World Bank, Washington, DC. World Bank. https://openknowledge.worldbank.org/handle/10986/32538 License: CC BY 3.0 IGO. [↑](#footnote-ref-2)
